# Effects of social support on music performance anxiety among university music students: chain mediation of emotional intelligence and self-efficacy

**DOI:** 10.3389/fpsyg.2024.1389681

**Published:** 2024-09-17

**Authors:** Zhang Huawei, Hashem Salarzadeh Jenatabadi

**Affiliations:** ^1^Department of Music, Dongchang College, Liaocheng University, Liaocheng, Shandong, China; ^2^Department of Science and Technology Studies, Faculty of Science, Universiti Malaya, Kuala Lumpur, Malaysia

**Keywords:** music performance anxiety, social cognitive theory, musical self-efficacy, emotional intelligence, social support, Stimulus-Organism-Response theory

## Abstract

The primary goal of this research is to investigate the relationship between social support and music performance anxiety in the context of social cognitive theory and stimulus-organization-response theory among music university students in China. The study involved both postgraduate and undergraduate students. The suggested framework consisted of three independent variables: parental support, teacher support, and peer support, two mediators: emotional intelligence and self-efficacy, and one dependent variable: music performance anxiety. A survey of 483 students was undertaken, and the data was analyzed using path analysis and structural equation modeling. The study discovered that all three forms of parental, teacher, and peer support were positively associated to both self-efficacy and emotional intelligence, with postgraduate students experiencing the strongest effects. Furthermore, self-efficacy and emotional intelligence had a negative significant effect on music performance anxiety, with the association being stronger among postgraduate students. Finally, self-efficacy and emotional intelligence emerged as significant mediators of the relationship between social support and music performance anxiety. These results add to our knowledge of the mechanisms by which social support influences music performance anxiety.

## 1 Introduction

Music performance anxiety is a complex condition that affects musicians of all skill levels, from beginners to experienced pros. It refers to a variety of emotional and physical symptoms that arise before or during a musical performance, frequently accompanied by feelings of uneasiness, dread, or terror (Lee, 2022). These emotions might be produced by the stress of being assessed, the dread of making mistakes, or the high expectations placed on oneself or others. Unlike normal anxiousness, music performance anxiety can be crippling, impairing a musician’s ability to perform effectively (Gómez-López and Sánchez-Cabrero, 2023; Sims and Ryan, 2023). It presents itself in a variety of ways, including shaking hands, racing heart, parched mouth, memory lapses, breathing symptoms, and a general feeling of panic (Guyon et al., 2020a; Osborne et al., 2014; Zakaria et al., 2013). These symptoms not only damage the quality of performance but can also lead to a negative spiral of anxiety and reduced self-confidence (Guyon et al., 2020b; Herman and Clark, 2023).

The fundamental causes of music performance anxiety are complex and can vary greatly from person to person. Perfectionism, low self-esteem, and a negative self-image are all common contributing factors (Butković et al., 2022; Sickert et al., 2022). Lupiáñez et al. (2022) and Sims and Ryan (2023) believed that fear of being judged negatively by an audience or peers is another important element to consider. Furthermore, a lack of preparation or prior unpleasant performance experiences might exacerbate nervousness (Spahn et al., 2021). For some, music performance anxiety is associated with bigger concerns like generalized anxiety disorder (MacAfee and Comeau, 2023). The physiological responses associated with music performance anxiety, including elevated heart rate and adrenaline production, are part of the body’s natural fight-or-flight response, which, while acceptable in stressful conditions, can be overpowering and counterproductive in a performance atmosphere.

Managing music performance anxiety necessitates a diverse strategy. Behavioral strategies like as systematic desensitization, in which the musician gradually exposes themselves to performance settings in a controlled and stepwise manner, can be useful. Cognitive techniques, such as cognitive-behavioral therapy (CBT), can assist change negative thought patterns and performance attitudes (Candia et al., 2023). Physical relaxation practices such as deep breathing, yoga, and mindfulness meditation can also help manage anxiety’s physical effects. Practical actions such as careful preparation, mock performances, and focusing on the pleasure of music rather than the dread of being judged can also be beneficial. It’s critical for musicians to understand that some anxiousness is normal and can even improve performance by raising energy and concentration (Juncos et al., 2017). Seeking support from instructors, colleagues, or mental health specialists can also help you manage music performance anxiety effectively.

Music performance anxiety is a widespread problem among music students, and it can greatly impair their ability to perform and enjoy their art. Understanding and addressing the factors that lead to music performance anxiety can help students manage and lessen their anxiety. Here are some ways and considerations:

### 1.1 Self-efficacy in music concept

Self-efficacy is an individual’s belief in their ability to successfully perform and complete music-related tasks, such as playing an instrument, singing, creating, or participating in musical performances. It is a subset of the broader psychological concept of self-efficacy, which was created by psychologist Albert Bandura and is described as confidence in one’s ability to organize and execute the courses of action required to manage certain scenarios (Bandura, 2001). Self-efficacy in music concept, or a musician’s conviction in their ability to successfully perform and achieve musical goals, has a substantial impact on their entire experience with music performance anxiety. Personal influences, such as good performances or constructive practice sessions, contribute to the development of self-efficacy in music, and external feedback from teachers, colleagues, and audiences influences this (González et al., 2018). High levels of self-efficacy are typically associated with lower levels of performance anxiety. Musicians who believe in their abilities see performances as opportunities to demonstrate their abilities, rather as challenges to their competence or self-esteem (Arbinaga, 2023). This positive outlook not only reduces anxiety, but it also improves performance overall. In contrast, musicians with poor self-efficacy frequently doubt their talents, which causes heightened tension and worry. They may consider difficult musical tasks as beyond their skills, creating a cycle of anxiety and avoidance that can harm both performance quality and personal growth.

The relationship between self-efficacy and performance anxiety is also influenced by the musician’s thinking and coping mechanisms (MacAfee and Comeau, 2020). Musicians with a growth mindset, who see setbacks as opportunities for learning and progress, have greater levels of self-efficacy (Harpaz and Vaizman, 2023). They are more likely to adopt effective practice tactics, solicit feedback, and apply constructive criticism to improve. These acts strengthen their conviction in their ability to achieve, resulting in a positive feedback loop that reduces music performance anxiety. On the other hand, musicians with a fixed mindset, who feel their abilities are intrinsic and unchangeable, may shun difficult pieces or performance opportunities out of fear of failing (Paese and Egermann, 2024). This avoidance might result in a lack of experience and progress, which reduces self-efficacy and increases anxiety. Coping tactics including positive self-talk, visualization, and goal setting can also boost self-efficacy, allowing musicians to perform with confidence and resilience.

### 1.2 Emotional intelligence in the music concept

Emotional intelligence in the music concept is a musician’s capacity to perceive, process, and convey emotions through music (Resnicow et al., 2004). This type of emotional intelligence is unique to the musical world and includes numerous important components. First, it entails identifying and understanding emotional content in music, both in terms of composition and performance (Chirico et al., 2015). Musicians with high emotional intelligence may detect subtle emotional nuances in compositions and effectively communicate them to the audience. Second, it encompasses the ability to use music as a means of expressing one’s own emotions, allowing musicians to incorporate their emotions into their performances (Sarkar et al., 2020). This makes their performance more honest and emotionally impactful. Finally, emotional intelligence entails empathizing with the audience, recognizing their emotional reactions, and tailoring the performance accordingly (Rakei et al., 2022). As a result, emotional intelligence linked to parental support enhances the ability to feel and express empathy toward colleagues during musical ensemble performances, fostering a collaborative and supportive environment. This skill is essential for engaging and connecting with listeners at a deeper level.

The link between emotional intelligence and music performance anxiety is nuanced and diverse. On one hand, artists with strong emotional intelligence may be more sensitive to performance anxiety due to their heightened sensitivity to emotional nuances in music and audience reactions (Kaleńska-Rodzaj, 2023). They may suffer heightened emotional responses to the notion of being harshly criticized for failing to portray the intended emotions in their performance. This sensitivity can lead to greater anxiousness and anxiety in anticipation of or during performances. On the other hand, emotional intelligence can also give artists with methods to control and lessen performance anxiety (Kaleńska-Rodzaj, 2021). For example, musicians can utilize their understanding of emotions to recognize and regulate their own anxiety-related symptoms. They can also employ music itself as a technique of coping, directing their nervous energy into their performance to enhance emotional expressiveness (Huang and Yu, 2022). This capacity to moderate emotions can lead to a more controlled and confident performance.

Furthermore, emotional intelligence influences how artists comprehend and react to the performance circumstances. Musicians with a high level of intelligence are better able to perceive audience reactions in a more balanced manner, lowering the possibility of interpreting the audience as too critical or unresponsive (Kaleńska-Rodzaj, 2020). They can also use their emotional talents to establish a stronger rapport with the audience, reducing nervousness. The ability to emotionally connect with the music and the audience can turn the performance experience from a stressful occasion to one of emotional expression and conversation. Educational initiatives aimed at fostering emotional intelligence, such as emotional awareness training in music, performance practice with emotional intent, and audience interaction techniques, can be useful (Kaleńska-Rodzaj, 2021). These tactics not only improve the musicality of performances, but also provide musicians with essential tools for dealing with the emotional issues that come with it, such as performance anxiety.

### 1.3 Social supports

The different kinds of help and support that people or groups give to each other, especially when they are going through a tough time, is called social support. It includes things like empathy, caring, love, and trust, as well as instrumental support like physical help and service, informational support like advice, ideas, and facts, and appraisal support like positive feedback and reassurance. Support like this can come from family, friends, teachers, leaders, or people in the community. Social support is an important part of mental health because it helps people deal with stress, feel like they belong, boost their self-esteem, and make them stronger against mental health problems.

Social support is extremely important for music students. Emotional support from family, friends, and teachers can increase students’ morale, especially when confronted with challenges such as mastering difficult pieces or dealing with performance anxiety (Herman and Clark, 2023). Instrumental support, such as financial aid for lessons or equipment, eliminates barriers to learning and development (Berg et al., 2022). Teachers and mentors frequently provide informational support to students, guiding them through the technical and theoretical parts of music. Finally, assessment support, which can be from peers, professors, or audiences, provides constructive feedback that helps students improve their talents and acquire a critical awareness of their art (Suzuki and Pitts, 2023). This diverse support network not only helps to develop musical skills, but it also offers a loving environment that can instill a lifelong interest and devotion to music.

Parental support is critical to how a music student copes with and controls performance anxiety. The emotional atmosphere created by parents can have a considerable impact on a child’s approach to musical performances (Kenny and Holmes, 2018). When parents create a supportive and understanding environment, it helps to boost the student’s self-esteem and lowers the fear of rejection or failure. This emotional support is especially important in music, since public performances can feel deeply personal. Parents who encourage constant practice, recognize efforts, and celebrate improvement, regardless of performance outcomes, aid in the development of a growth attitude (Barnes et al., 2016). This technique turns the emphasis from fear of failure to appreciation of learning and self-improvement, reducing performance anxiety. Furthermore, parents who respond calmly and positively to stress and challenges can teach their children similar coping skills, allowing them to better manage their nerves during performances (Huang and Yu, 2022).

Teachers have a significant impact on their students’ experiences with musical performance anxiety. A teacher’s teaching style and classroom atmosphere can greatly reduce or worsen performance anxiety in students. Teachers who prioritize skill development, provide constructive and supportive feedback, and foster a safe, non-judgmental learning atmosphere can significantly boost a student’s confidence (Tahirbegi, 2022). This confidence is critical in lowering performance anxiety. Furthermore, teachers can directly address performance anxiety by including tactics like simulated performance experiences, relaxation and breathing exercises, and positive imagery into their instructional approaches (Barros et al., 2022). By doing so, they assist students in developing not only musical skills, but also the mental and emotional tools required to deal with the stresses of performing. Furthermore, teachers can foster a culture of mutual support and understanding among students, thereby establishing an environment in which students feel safe to share and work on their fears (MacAfee and Comeau, 2023).

Peer support is also important in managing anxiety associated with music performance. The support of fellow students fosters a sense of camaraderie and shared experience, which is important in dealing with performance-related stress and anxiety (Huang and Yu, 2022; McGrath et al., 2016). When students witness their friends dealing with similar issues, they feel less alone and more understood in their experiences. This social support can take many forms, including encouragement, sharing effective coping skills, or simply lending a sympathetic ear (Schletter, 2020). Importantly, comments and advice from peers can be more relatable and less daunting than that from adults, making it an essential component in overcoming performance anxiety. Furthermore, peer interactions, such as group practices or ensemble performances, allow students to become accustomed to performing in front of others in a less formal and more supportive environment, which can assist to gradually reduce performance-related stress (Biasutti and Concina, 2014).

### 1.4 Level of education

Educational level is a core term in educational studies that represents the many stages of learning and cognitive development. In common educational environments, these stages are usually classified as primary, secondary, and higher education. Each stage is targeted to the children’s ages and developmental stages, with curricula designed to gradually introduce and build on skills and knowledge. Primary education frequently focuses on foundational skills such as literacy and basic mathematics, as well as an introduction to more general courses such as science, history, and the arts. The goal here is to develop a broad base of knowledge and instill a passion for studying. As students go through secondary school, the emphasis shifts to greater investigation of subjects, with a concentration on critical thinking, analysis, and specialized knowledge. Meanwhile, higher education entails advanced study in certain subjects, which promotes autonomous thought, research, and knowledge application. This hierarchical structure ensures that students receive a thorough educational experience, providing them with the skills and knowledge they need for personal and professional success.

In the context of music education studies, the educational levels follow a similar pattern, but with a distinct emphasis on both technical proficiency and creative expression. Undergraduate music education often comprises fundamental theory, introduction to many forms of music, and basic singing or instrument practice (Hoffer, 2017). This level tries to instill a fundamental grasp of music and an appreciation for it. As music students progress through postgraduate level, their music education becomes more rigorous and specialized, sometimes incorporating advanced theory, intensive instrument or vocal training, and involvement in groups or choirs (Conway, 2020). The curriculum aims to improve students’ technical ability while also instilling a sense of musical history and context. Higher music education, such as master and PhD programs, places an even greater emphasis on specialized, in-depth study of a certain area of music (Conway, 2020). In related music performance anxiety studies, several researchers focused on only undergraduate or postgraduate students. It means that in the context of music performance anxiety, combining undergraduate and postgraduate data in a single dataset is not an appropriate method for analysis and modeling.

### 1.5 This study

The current study uses the integration of SOR (Stimulus-Organism-Response) theory (Mehrabian and Russell, 1974) and social cognitive theory (Bandura, 1978) as an integrated theory to explain students’ anxiety issues during music performances. The SOR model is a concept used in environmental psychology to better understand how people react to external stimuli. In this approach, a stimulus (S) is any external factor that can evoke a response, such as a physical setting, a social scenario, or a promotional message. The organism (O) symbolizes the individual who sees and processes the input, with internal influences such as emotions, attitudes, and cognitive processes. The response (R) is the individual’s reaction or behavior caused by the interplay of the stimulus and their internal state. This approach emphasizes the significance of internal processing in influencing how an individual reacts to external stimuli (Mehrabian and Russell, 1974). Self-efficacy has an impact on an individual’s music performance anxiety within the framework of Social Cognitive Theory.

This study takes parental, teacher, and peer support as a stimulus, self-efficacy and emotional intelligence as an organism, and music performance anxiety as a response. The study also takes into account students’ concerns about music performance anxiety at two levels of education: undergraduate and postgraduate and compares the results of their models. Given this, the current study attempts to evaluate the following:

1. Relationships of parental support with (a) self-efficacy and (b) emotional intelligence at two levels of education: undergraduate and postgraduate among music students.
2. Relationships of teacher support with (a) self-efficacy and (b) emotional intelligence at two levels of education: undergraduate and postgraduate among music students.
3. Relationships of peer support with (a) self-efficacy and (b) emotional intelligence at two levels of education: undergraduate and postgraduate among music students.
4. The relationship between (a) self-efficacy and (b) emotional intelligence and music performance anxiety in two undergraduate and postgraduate music students.
5. The mediating roles of self-efficacy and emotional intelligence between (a) parental support, (b) teacher support, (c) peer support and music performance anxiety.

**Figure 1:**
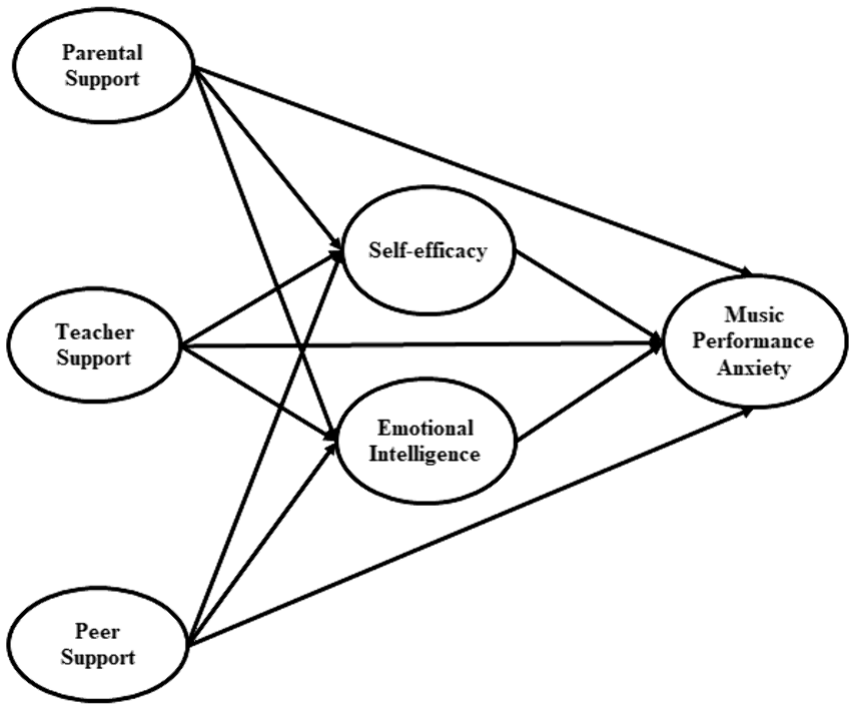
Research model.

The proposed model is depicted in Figure 1.

## 2 Materials and methods

### 2.1 Statistical method

For this study, we used Structural Equation Modeling (SEM) as the main statistical tool, and we analyzed the data using the AMOS software. SEM is a powerful statistical method that can analyze intricate connections between observed and underlying variables. It enables the assessment of both direct and indirect effects inside the model. SEM allows for the evaluation of the extensive interplay of variables such as parental support, teacher support, peer support, self-efficacy, emotional intelligence, and music performance anxiety. AMOS enhanced this procedure by offering sophisticated modeling capabilities, visual depiction of the models, and rigorous statistical analysis, guaranteeing the dependability and accuracy of our discoveries.

### 2.2 Measures

In SEM, adequate measurement of the research variables is critical for model correctness and interpretability. The procedure begins by operationalizing latent variables, which are theoretical constructs that are not directly observable, using several observed indicators or measurement variables. This operationalization is informed by theoretical understanding and prior research, ensuring that the indicators used are valid and reliable representations of the hidden constructs. In this study, we have six latent variables, which are parental support, teacher support, peer support, self-efficacy, emotional intelligence, and music performance anxiety (see Table 1). The research variables were assessed using a Likert scale with a range of 1–7 in this study. The participants were given a scale to indicate their level of agreement or disagreement with items relating to the constructs being examined, including parental support, teacher support, peer support, self-efficacy, emotional intelligence, and music performance anxiety. A score of 1 denotes a significant level of disagreement, while a score of 7 signifies a substantial level of agreement.

**Table 1:** Theoretical support for measurement variables.

Latent Variable	Quantity of inquiries	Theoretical support
Parental support	5 items	Ryan et al. (2000)
Teacher support	5 items	Ryan et al. (2000)
Peer support	5 items	Ryan et al. (2000)
Music self-efficacy	12 items	Ritchie and Williamon (2011)
Emotional intelligence	16 items	Wong and Law (2002)
Kenny Music Performance Anxiety Inventory (K-MPAI)	26 items	Kenny et al. (2004)

#### 2.2.1 Music performance anxiety

The Kenny Music Performance Anxiety Inventory (K-MPAI) is a specialized evaluation tool created by Kenny et al. (2004) that measures music performance anxiety in both amateur and professional musicians. This inventory marks a big step forward in the field of music psychology, meeting the demand for a comprehensive, psychometrically sound measure of performance anxiety specific to musicians. The K-MPAI is based on significant research into the various aspects of performance anxiety, as well as theoretical models that incorporate cognitive, behavioral, and physiological components of anxiety. Its framework normally consists of a sequence of items that respondents score based on their experiences and opinions about music performance. The checklist is intended to capture a broad range of anxiety symptoms and triggers, including cognitive features like fear of unfavorable appraisal, emotional symptoms like anxiousness or dread, and bodily symptoms like trembling or sweating.

The K-MPAI is unusual in that it takes a multidimensional approach to music performance anxiety, taking into account a variety of contributing elements. It evaluates not just the current symptoms experienced during performances, but also more general characteristics that can influence anxiety, such as personality qualities, past experiences, and situational considerations (Dias et al., 2022). For example, it might look into perfectionism, a musician’s performance history, and performance context (e.g., solo vs. ensemble, audience kind). The K-MPAI is useful for both research and practical purposes. It aids studies in determining the prevalence, causes, and effects of music performance anxiety, hence leading to a better knowledge of the issue (Bellinger et al., 2023). In practical situations such as music education and therapy, the K-MPAI can be used to identify individuals who suffer considerably from performance anxiety, leading to interventions and support measures. This inventory is especially important for music educators, therapists, and performers, as it provides insights into how to better manage music performance anxiety and improve overall performance experiences.

There was substantial factor loadings found in each of the survey questions that were supposed to measure the music performance anxiety construct. These factor loadings ranged from 0.731 to 0.791. It was determined that the construct reliability of the scale was 0.787, which is significantly higher than the permissible threshold of 0.60. This fact indicates that the scale has a high degree of internal consistency. In addition, the Average Variance Extracted (AVE) was 0.611, which is higher than the minimum threshold of 0.5. This demonstrates that the scale has a high level of discriminant and construct validity. It is also noteworthy that the goodness of fit indices of the scale were rather outstanding, with a Goodness of Fit Index (GFI) value of 0.923, an Adjusted Goodness of Fit Index (AGFI) value of 0.931, a Normed Fit Index (NFI) value of 0.988, an Incremental Fit Index (IFI) value of 0.933, and an Root Mean Square Error of Approximation (RMSEA) value of 0.036. These findings collectively imply that the scale is a trustworthy and valid instrument for assessing anxiety related to performing music, as it correlates closely with the data and offers measurements that are consistent.

#### 2.2.2 Self-efficacy

Ritchie and Williamon (2011) study on self-efficacy (in musical concept) presented a specific scale for measuring musicians’ confidence in their ability to do music-related activities. This scale includes remarks about important areas of musical performance, such as technical capabilities, interpretative talents, and performance under duress.

The study of these replies offers a complete picture of a musician’s self-efficacy. High scores typically indicate a strong belief in one’s own musical ability, which is frequently associated with improved performance outcomes and increased motivation. Lower ratings, on the other hand, may show areas where a musician lacks confidence, which could lead to focused interventions or support. This scale, confirmed through empirical study, is especially beneficial for music instructors and academics to understand and improve musicians’ performance by addressing their self-beliefs (Dias et al., 2022).

The factor loadings of the survey items that were designed to measure the construct of self-efficacy ranged from 0.709 to 0.821, which indicates that there are high correlations with the underlying construct. A construct reliability of 0.711 was found for the scale, which was higher than the evaluation criterion of 0.60, so showing that the scale possessed internal consistency. Furthermore, the Average AVE was 0.641, which is higher than the minimal requirement of 0.5. This demonstrates that the scale possesses excellent discriminant and construct validity by exceeding the minimum requirement. Furthermore, the robustness of the scale was validated by the goodness of fit indices, which comprised a GFI of 0.909, an AGFI of 0.919, an NFI of 0.921, an IFI of 0.976, and an RMSEA of 0.021. In light of these findings, it can be concluded that the scale is a valid and trustworthy instrument for evaluating self-efficacy, since it demonstrates a strong match to the data.

#### 2.2.3 Emotional intelligence

The Wong and Law Emotional Intelligence Scale (WLEIS) is a self-report measure created by Wong and Law (2002) to examine the concept of emotional intelligence in individuals. This scale is based on the premise that emotional intelligence, or the ability to recognize, comprehend, use, and manage one’s own and others’ emotions, is critical for personal and societal functioning. The WLEIS is made up of 16 items that are systematically separated into four subscales, each representing a significant characteristic of emotional intelligence: self-emotion appraisal (SEA), others’ emotion appraisal (OEA), use of emotion (UOE), and regulation of emotion (ROE). These factors assess an individual’s ability to understand and express their own emotions, recognize and interpret emotions in others, harness emotions to aid cognitive processes, and manage emotions to promote emotional and intellectual development (Balti and Karoui Zouaoui, 2024).

With factor loadings ranging from 0.709 to 0.802, the survey questions that were supposed to measure the emotional intelligence construct showed significant factor loadings. This indicates that the survey questions have high connections with the underlying construct. It was determined that the construct dependability of the scale was 0.739, which is significantly higher than the acceptable threshold of 0.60, indicating that the scale had a high level of internal consistency. In addition, the AVE was 0.588, which is higher than the minimum threshold of 0.5. This demonstrates that the scale has strong discriminant and construct validity. In addition, the goodness of fit indices of the scale were rather remarkable, sporting a GFI value of 0.913, an AGFI value of 0.925, an NFI value of 0.976, an IFI value of 0.919, and an RMSEA value of 0.021. Collectively, these findings suggest that the scale is a robust and trustworthy instrument for evaluating emotional intelligence. It aligns closely with the data and provides measurements that are consistent with one another.

#### 2.2.4 Social support

Ryan et al. (2000) introduced the Social Support Scale in 2000, which is a psychological instrument used to assess perceived social support in diverse life settings. This scale, developed within the framework of self-determination theory, highlights the role of social support in promoting psychological growth, intrinsic drive, and well-being (Zarza-Alzugaray et al., 2020). The scale primarily measures an individual’s perception of their social environment as helpful, compassionate, and encouraging of autonomy. It is based on the concept that social support is more than just the presence of people; it is also about the quality of interpersonal relationships and how well these connections meet core psychological needs like autonomy, competence, and relatedness (Orejudo et al., 2021).

The Social Support Scale normally includes multiple items or phrases that respondents score to reflect their perceptions of support in various relationships, such as those with parents, teachers, and peers. The scale measures many aspects of support, such as emotional support (the degree to which people feel cared for and loved), instrumental support (the availability of practical assistance), and informational support (access to counsel and direction). It may also assess the degree of autonomy support, which is the extent to which a person’s social environment encourages them to make their own decisions and pursue their interests. Ryan et al. (2000) developed the Social Support Scale, which has been widely used in psychology research to investigate the association between social support and various outcomes such as mental health, motivation, and overall well-being. Its emphasis on autonomy support makes it especially applicable in circumstances where self-determination and personal growth are critical, such as education, the workplace, and therapeutic interventions.

Each of the survey questions that were designed to evaluate the social support construct exhibited significant factor loadings, with values ranging from 0.719 to 0.819. This suggests that there is a strong alignment with the construct that is being discussed. The construct reliability of the scale was 0.798, which resulted in a considerable increase above the evaluation criterion of 0.60, so emphasizing the scale’s excellent level of internal consistency. In addition, the AVE was 0.539, which was higher than the minimum criterion of 0.5. This provides further evidence that the measure possesses both strong construct validity and great discriminant validity. There was also remarkable goodness of fit indices displayed by the scale, including a GFI of 0.919, an AGFI of 0.937, a NFI of 0.918, an IFI of 0.944, and a CFI of 0.909. Furthermore, the Root Mean Square Error of Approximation (RMSEA) was found to be 0.031. These indices, when taken as a whole, indicate that there is a strong fit between the model that was proposed by the scale and the data that was seen. When taken as a whole, these findings suggest that the scale is a robust and trustworthy instrument for assessing social support. Not only does it align well with the data, but it also measures the construct that it intends to evaluate in a consistent manner. This makes it a reliable instrument for research and practical applications in the field of psychology.

### 2.3 Main data collection

A power analysis was performed using the G*Power software, which revealed that the inquiry requires a minimum sample size of 466 individuals (see Supplementary material). The computation was performed using an expected effect size of 0.15, a predetermined alpha value of 0.05, and an estimated power of 0.85. The questions were initially written in English and then thoroughly evaluated by two professionals who are fluent in both Chinese and English. This approach required using a translation and then retranslation technique to ensure accuracy. A total of 500 paper-and-pencil questionnaires were distributed to participants, with 483 successfully collected, for a response rate of 96.6%.

## 3 Results

### 3.1 Descriptive statistics and correlation analysis

Out of the replies gathered, 47% were classified as male, while 53% were classified as female. The sample comprised music students, and their distribution was as follows: 51.3% of the students were categorized as undergraduate students, while 48.7% were classified as postgraduate students. The age distribution of the participants was as follows: The age cohort of individuals aged “less than 22 years old” constituted 29.6% of the sample, while the age group spanning from 22 to 28 years represented 34.6% of the participants. Among the respondents, 23.0% belonged to the age range of 29–32 years, while persons who were categorized as “older than 32 years old” made up 12.8% of the sample. The descriptive statistics of the constructs are given in Table 2.

**Table 2:** Descriptive statistics of constructs.

Construct	Mean	SD	95% CIs
Parental support (5 items)	5.25	1.29	[3.96, 6.54]
Teacher support (5 items)	5.27	1.28	[3.99, 6.55]
Peer support (5 items)	5.19	1.31	[3.88, 6.50]
Self-efficacy (12 items)	5.27	1.26	[4.01, 6.53]
Emotional intelligence (16 items)	5.25	1.30	[3.95, 6.55]
Music performance anxiety (26 items)	5.17	1.31	[3.86, 6.48]

The correlation analyses are given in Table 3.

**Table 3:** Correlation analysis.

Undergraduate						
	(1)	(2)	(3)	(4)	(5)	(6)
(1) Parental support	1					
(2) Teacher support	0.087	1				
(3) Peer support	0.037	0.065	1			
(4) Musical self-efficacy	0.476	0.511	0.387	1		
(5) Musical emotional intelligence	0.442	0.498	0.404	0.035	1	
(6) Music performance anxiety	−0.376	−0.553	−0.369	−0.439	−0.609	1

Postgraduate						
	(1)	(2)	(3)	(4)	(5)	(6)
(1) Parental support	1					
(2) Teacher support	0.063	1				
(3) Peer support	0.032	0.041	1			
(4) Musical self-efficacy	0.463	0.489	0.337	1		
(5) Musical emotional intelligence	0.426	0.465	0.386	0.062	1	
(6) Music performance anxiety	−0.321	−0.517	−0.323	−0.487	−0.667	1

### 3.2 Validity and reliability

The notions of validity and reliability are critical in SEM because they ensure the correctness and consistency of the measurement model, which is a component of SEM. In SEM, validity refers to how effectively the model represents the theoretical constructs it is supposed to measure. Reliability, on the other hand, refers to the consistency of the measurement model, which ensures that the latent variables are accurately measured by their indicators.

Fornell and Larcker (1981) define a set of conditions that must be completed in order to assess the validity and reliability of a survey using SEM. To be deemed genuine, a latent variable must have a Cronbach’s alpha coefficient of at least 0.7. Table 2 demonstrates that the Cronbach’s alpha values for each latent variable meet the established standards, implying that this study is legitimate. Furthermore, Average Variance Extracted (AVE) is a widely used statistic for determining reliability. According to Segars (1997), in order to receive reliability clearance, the value of this index should be greater than 0.5. This indicator satisfies the required ideas and criteria. Thus, the study model’s reliability has been proven.

### 3.3 Common method variance

In SEM, common method variance (CMV) refers to the variance that may be attributed to the measuring method rather than the constructs represented by the measures. This issue develops when both independent and dependent variables are collected using the same approach, resulting in artificially inflated or deflated correlations. In SEM, CMV can undermine the validity of inferred correlations between constructs by adding systematic inaccuracy. Moreover, CMV is the discrepancy between two sets of data induced by the measurement method rather than the things being measured. This could be a difficulty in behavioral research, particularly when a single method (such as a survey) is used to test multiple domains. To limit variation, we collected anonymous data and scored some items backwards. After gathering the data, Harman’s univariate factor analysis was utilized to see whether there was any common-method variance. There were 12 factors with eigenvalues greater than one. The first factor accounted for 15.3% of the variation, less below the necessary standard of 40%. This clearly shows that the data in this study have no significant common-method variance.

### 3.4 Model fitting

Model fitting in SEM determines how well a given model describes the data. This procedure is critical for determining the model’s validity and usefulness in understanding the links between observable and latent variables. SEM evaluates model fit using a variety of fit indices and statistical tests. Absolute fit metrics include the Chi-square test, goodness-of-fit index (GFI), and root mean square error of approximation (RMSEA), whereas relative fit measures include the comparative fit index (CFI) and Tucker-Lewis index (TLI). Additionally, incremental fit measurements such as the normed fit index (NFI) are utilized. Each of these indices provides unique information about the model’s fit, with lower RMSEA values and higher CFI and TLI values indicating a better match. A well-fitting model is one in which the hypothesized model structure accurately matches the observed data structure.

According to the findings of Byrne (2013) and Kline (2010), it is recommended that a research model have fit values greater than 0.9. The results of the fitting analysis indicate that the goodness of fit indices, such as GFI, RFI, IFI, TLI, CFI, and NFI, for both undergraduate and postgraduate groups are all higher than the acceptable threshold of 0.90, suggesting a strong fit for the model. The indices for students are as follows: GFI (0.927), RFI (0.905), IFI (0.933), TLI (0.924), CFI (0.918), and NFI (0.916). The numbers for postgraduates are as follows: GFI (0.918), RFI (0.918), IFI (0.923), TLI (0.905), CFI (0.912), and NFI (0.901). In addition, the RMSEA values for undergraduates and postgraduates are 0.032 and 0.028 respectively, which are both inside the acceptable range (less than 0.05). This provides further evidence of a high fit for the model. These findings indicate that the model is suitable for both educational levels, indicating that the constructs assessed are reliable and valid across various educational settings.

### 3.5 Structural model

The structural model is an important component of SEM because it captures the predicted links between latent variables (unobserved constructs) and, in some situations, between latent and observed variables. This model, frequently represented graphically as a path diagram, is made up of a set of regression-like equations in which latent variables are linked by directional routes to reflect causal hypotheses. These pathways’ coefficients measure the intensity and direction of the relationships.

Table 4 shows the results of SEM for undergraduate and postgraduate students. Parental, teacher, and peer support have been shown to have a significant positive relationship with self-efficacy and emotional intelligence among undergraduate and postgraduate students. Both self-efficacy and emotional intelligence have a strong relationship with music performance anxiety. For both undergraduate and postgraduate students, teacher support has a greater influence on music performance anxiety than parental and peer support do. The correlation between self-efficacy and emotional intelligence is stronger in postgraduate students than in undergraduates. Furthermore, self-efficacy and emotional intelligence have a greater impact on music performance anxiety in postgraduate students than in undergraduate students.

**Table 4:** Direct and indirect effect.

Relationship	Estimated standardized coefficients	LL 95% CI	UL 95% CI	Estimated standardized coefficients	LL 95% CI	UL 95% CI
	Undergraduate students (*n* = 248)			Postgraduate students (*N* = 235)		
Direct effect						
Parental support → Self-efficacy	0.562***	0.501	0.612	0.463***	0.429	0.497
Parental support → Emotional intelligence	0.487***	0.449	0.509	0.426***	0.394	0.467
Parental support → Music performance anxiety	−0.389**	−0.407	−0.338	−0.321**	−0.358	−0.296
Teacher support → Self-efficacy	0.555***	0.491	0.583	0.489***	0.423	0.519
Teacher support → Emotional intelligence	0.501***	0.478	0.521	0.465***	0.424	0.509
Teacher support → Music performance anxiety	−0.545***	−0.582	−0.533	−0.517***	−0.558	−0.483
Peer support → Self-efficacy	0.428**	0.373	0.441	0.337**	0.289	0.377
Peer support → Emotional intelligence	0.491***	0.463	0.516	0.386**	0.352	0.413
Peer support → Music performance anxiety	−0.311**	−0.331	−0.298	−0.323**	−0.387	−0.297
Self-efficacy → Music performance anxiety	−0.533***	−0.562	−0.489	−0.487***	−0.538	−0.444
Emotional intelligence → Music performance anxiety	−0.714***	−0.722	−0.681	−0.667***	−0.702	−0.626
Indirect effect						
Parental support → Self-efficacy → Music performance anxiety	−0.300*	−0.319	−0.244	−0.225*	−0.266	−0.216
Parental support → Emotional intelligence → Music performance anxiety	−0.348*	−0.361	−0.287	−0.284*	−0.333	−0.221
Teacher support → Self-efficacy → Music performance anxiety	−0.296*	−0.318	−0.231	−0.238*	−0.277	−0.219
Teacher support → Emotional intelligence → Music performance anxiety	−0.358*	−0.372	−0.308	−0.310**	−0.367	−0.228
Peer support → Self-efficacy → Music performance anxiety	−0.228*	−0.247	−0.198	−0.164*	−0.205	−0.113
Peer support → Emotional intelligence → Music performance anxiety	0.222*	0.172	0.235	0.215*	0.188	0.265

* < 0.05, ** < 0.01, *** < 0.001.

### 3.6 Multigroup analysis

In the study, where educational level serves as a moderator, the Wald test is particularly useful for analyzing whether the relationships between variables, such as social support, self-efficacy, emotional intelligence, and music performance anxiety, differ across educational levels (e.g., undergraduate versus postgraduate students). By applying the Wald test, it is possible to assess whether the effect of these factors on outcomes like music performance anxiety is moderated by educational level. If the Wald test reveals significant differences in the path coefficients between the two groups, it would indicate that educational level indeed plays a moderating role, meaning that the influence of factors like social support or self-efficacy varies depending on whether a student is an undergraduate or a postgraduate (Table 5).

**Table 5:** Multigroup analysis.

Relationships	$\mathrm{Wald}\;\chi^{2}$ test	*p*-value
Direct effects		
Parental support → Self-efficacy	6.60	0.018
Parental support → Emotional intelligence	5.45	0.021
Parental support → Music performance anxiety	−4.25	0.033
Teacher support → Self-efficacy	4.71	0.031
Teacher support → Emotional intelligence	4.00	0.039
Teacher support → Music performance anxiety	−1.5	0.370
Peer support → Self-efficacy	3.95	0.041
Peer support → Emotional intelligence	3.88	0.046
Peer support → Music performance anxiety	0.33	0.767
Self-efficacy → Music performance anxiety	−5.11	0.023
Emotional intelligence → Music performance anxiety	−3.91	0.049
Indirect effects		
Parental Support → Self-efficacy → Music performance anxiety	2.08	0.107
Teacher Support → Self-efficacy → Music performance anxiety	1.35	0.305
Peer support → Self-efficacy → Music performance anxiety	1.24	0.392
Parental support → Emotional intelligence → Music performance anxiety	0.94	0.551
Teacher support → Emotional intelligence → Music performance anxiety	1.9	0.227
Peer support → Emotional intelligence → Music performance anxiety	2.73	0.079

## 4 Discussion

This study looked to examine the relationship between three types of social support—parents, teachers, and peers—and music performance anxiety among Chinese music students. This study looked at the direct association between social support and both self-efficacy and emotional intelligence among music university students for the first time. In addition, the study sought to investigate the mediating functions of self-efficacy and emotional intelligence in the relationship between social support and music performance anxiety.

First, the study contributes to the body of research on music performance anxiety by identifying strong negative correlations between social support and music performance anxiety among Chinese music students at both the undergraduate and postgraduate levels.

Social Support: Social support, which includes encouragement and aid from parents, teachers, classmates, and the larger social network, is critical in reducing music performance anxiety. This assistance works as a buffer against the stress of performance. When artists feel supported, they feel more secure and understood, which helps to normalize their anxiety and lessens feelings of isolation (Herman and Clark, 2023). Positive reinforcement from these social groups can increase self-confidence, motivate individuals, and encourage a more relaxed and pleasurable approach to performance.

Teacher’s support: teachers and music instructors play an important role in influencing a student’s approach to performing and coping with nervousness. A teacher’s approach to education and feedback has a considerable impact on a student’s self-esteem and confidence (Tahirbegi, 2022). Constructive feedback that emphasizes improvement and learning, rather than criticism, can boost a student’s self-efficacy and reduce anxiety. Teachers play an important role in providing students with the required performance skills and coping methods (MacAfee and Comeau, 2023). This includes teaching ways for coping with physical symptoms of anxiety, such as breathing exercises, as well as cognitive strategies like positive self-talk and imagery. Furthermore, teachers can provide low-stress performance chances for students to practice and feel comfortable performing in front of others, gradually increasing their confidence and decreasing performance-related anxiety (Barros et al., 2022).

Parents support: parents play a diverse role in managing music performance, which includes emotional, motivational, and practical components. Emotionally supportive parents create a safe environment for young musicians to communicate their anxieties and fears without being judged, promoting a sense of comfort and understanding (Kenny and Holmes, 2018). This emotional support is critical because it normalizes the experience of anxiety and promotes open discussion about feelings, both of which are required for effective anxiety management (Huang and Yu, 2022). On a motivational level, parents can favorably impact their child’s attitude toward performance by emphasizing the joy and personal fulfillment that music provides, rather than focusing primarily on success or achievement. This method can help to change the focus from fear of failure to pure enjoyment of the musical experience. Parents can help by creating a positive practice environment, establishing consistent routines, and giving logistical assistance for performances (Barnes et al., 2016). Such practical assistance not only alleviates the physical strains placed on young musicians, but also reduces anxiety by decreasing external stressors associated with performance preparation.

The conceptual similarities between the items measuring parental support and self-efficacy in the KMPAI questionnaire have had a significant yet controllable effect on our findings. The presence of these overlaps likely enhanced the observed relationships between parental support and self-efficacy, as the domains are essentially interconnected. Parental support frequently boosts self-efficacy by offering emotional and motivational support, which subsequently impacts performance results. Although this interconnection can enhance specific correlations, it also mirrors real-world dynamics in which support structures are vital for the development of human competencies. In order to assure the strength and reliability of our results, we made careful to employ meticulous statistical techniques to differentiate the distinct influences of each factor. By recognizing and dealing with these areas of overlap, our goal is to offer a detailed comprehension of how parental support and self-efficacy collectively impact music performance anxiety. This acknowledgment enhances our conversation by emphasizing the intricate interaction between various types of assistance and individual capabilities, ultimately providing significant understanding into the aspects that influence performance results.

Peer support: peers can have a significant impact on a musician’s experience with performance anxiety, both favorably and adversely. Positive peer relationships, such as encouragement, shared experiences, and compassionate understanding, can foster a sense of camaraderie and belonging while lowering feelings of isolation and stress (Huang and Yu, 2022; McGrath et al., 2016). Knowing that others are encountering similar issues might help to normalize performance anxiety and provide opportunities to share coping strategies (Biasutti and Concina, 2014). In group performance contexts, such as orchestras or bands, a supportive peer group provides a collaborative and non-judgmental environment, which can considerably reduce the pressure to perform flawlessly (Schletter, 2020). On the other side, negative peer relationships, such as competition or criticism, can worsen anxiety (Yoder, 2022). Thus, developing a supportive and positive peer culture in musical situations is critical for reducing the impacts of performance anxiety.

Second, the study contributes significant findings to the self-efficacy and emotional intelligence literature in music performance anxiety research. Self-efficacy and emotional intelligence are two important characteristics that influence music performance anxiety. Self-efficacy, or an individual’s conviction in their capacity to successfully execute music performance tasks, has a significant impact on how they experience anxiety (Spahn et al., 2023). High levels of self-efficacy are often associated with lower performance anxiety, as confident musicians are less likely to doubt their abilities or dread negative feedback. Previous accomplishments, positive comments, and a comprehensive preparation process can all help to boost confidence. Musicians with high self-efficacy are more likely to see difficult performances as opportunities to demonstrate their abilities rather than as threats, which reduces anxiety (MacAfee and Comeau, 2020). In contrast, low self-efficacy can heighten anxiety because musicians may anticipate failure, obsess about potential mistakes, and feel less in control of the performance outcome.

Emotional intelligence, on the other hand, is the ability to sense, use, comprehend, and control emotions within the framework of musical activity. Musicians with high emotional intelligence are more likely to perceive and understand their own feelings, as well as those of others, such as fellow musicians and the audience (Kaleńska-Rodzaj, 2023). This understanding enables individuals to better navigate the emotional terrain of a performance. For example, Mazzon et al. (2023) believe that musicians could employ emotional intelligence to direct their nervous energy into a more passionate and engaging performance. Furthermore, emotional intelligence entails the ability to manage one’s emotions, which is essential for dealing with music performance anxiety. Musicians who can skillfully manage their emotions can keep regular performance anxieties from turning into crippling anxiety. Furthermore, the sympathetic understanding associated with emotional intelligence can build a supportive environment among peers, so lowering the stress and competitive pressure that frequently accompanies musical performances (Kaleńska-Rodzaj, 2021). Thus, both self-efficacy and emotional intelligence play important roles in defining the severity and impact of music performance anxiety, each contributing in unique but complimentary ways.

Third, this study takes a novel approach by using self-efficacy and emotional intelligence as chain mediators to investigate the relationship between social support and music performance anxiety. Previous research has shown that social support has a high association with both self-efficacy (Orejudo et al., 2021; Wang and Wong, 2022; Zarza-Alzugaray et al., 2020) and emotional intelligence (Antonini Philippe et al., 2022; Kaleńska-Rodzaj, 2020). Previous research has also shown that self-efficacy (MacAfee and Comeau, 2020; Spahn et al., 2023) and emotional intelligence (Kaleńska-Rodzaj, 2021, 2023; Mazzon et al., 2023) are linked to music performance anxiety. Furthermore, the relationship between self-efficacy and emotional intelligence is recognized not only in music performance research (Esteve-Faubel et al., 2021; Kaleńska-Rodzaj, 2023; van Rensburg, 2005), but also in other educational studies (Mercader-Rubio et al., 2023; Pilotti et al., 2023). In this study, we discovered that self-efficacy and emotional intelligence mediate the relationship between music performance anxiety and social support from parents, teachers, and peers. We introduced a sophisticated framework, as seen in Figure 1. Prior research has not primarily focused on examining either self-efficacy or emotional intelligence as a mediator between social support and music performance anxiety.

Last, the study has revealed that the effect of social support on both self-efficacy and emotional intelligence is higher among undergraduate students in China compared to postgraduate students, based on multigroup analysis (see section 3.6). Nevertheless, the impact of both self-efficacy and emotional intelligence on the music performance anxiety of postgraduate students is stronger in comparison to undergraduate students. Undergraduate students are often at an earlier developmental stage than postgraduate students. They are frequently still developing their identities and self-perceptions, including their musical identities. As a result, the support they receive from their parents, teachers, and classmates may have a greater impact on their self-efficacy and emotional intelligence (Morales-Rodríguez and Pérez-Mármol, 2019). Positive praise and support at this point can help them gain confidence and emotional skills. In contrast, postgraduate students are often more mature and may have a stronger sense of self and self-efficacy, making them less vulnerable to external influences.

Undergraduate students typically have less experience and are still learning the ropes of their musical path. They may rely more heavily on external validation and assistance to boost their confidence and emotional coping abilities. In this situation, social support is critical to their development. Postgraduate students, on the other hand, frequently have more experience and have established a degree of independence in their musical abilities (Long et al., 2014). To overcome performance anxiety and emotional issues, they may rely on internal resources and past experiences rather than social assistance.

The academic environment and expectations for undergraduate and postgraduate students can differ significantly. Undergraduates are frequently enrolled in more structured programs with more regular and diverse performance chances, and social support can have a direct impact on their confidence and emotional management (Frantz et al., 2022). Postgraduates may confront a variety of pressures, including specialized performances, research, or teaching obligations, and their coping techniques may be more internally motivated as a result of their advanced training and expertise.

### 4.1 Practical implications

The effects of social support, which includes parents, teachers, and peers, on music performance anxiety, chain mediated self-efficacy, and emotional intelligence have important practical implications for educators, university officials, and students themselves. Gaining a thorough grasp of these implications can lead to the creation of more effective methods for reducing anxiety during their music performance. Here are a few important practical consequences:

- Parents can play an important part in developing their children’s confidence and emotional skills. They can increase their child’s self-efficacy by encouraging him or her and showing appreciation toward their efforts. This assistance encourages children to believe in their abilities to succeed in musical tasks, lowering performance anxiety. Parents can also demonstrate and teach emotional intelligence abilities such as empathy, emotional management, and constructive emotional expression. This can be accomplished by having open conversations about emotions, fostering introspection on emotional experiences associated with music, and demonstrating understanding and support during times of performance-related stress.
- Music professors and instructors have a direct impact on their pupils’ musical confidence and emotional intelligence. They can accomplish this by fostering a pleasant and supportive learning atmosphere in which mistakes are viewed as part of the learning experience. This method aids in lowering the dread of unfavorable evaluation, which is a major element in performance anxiety. Teachers can also offer specialized training in performance skills and anxiety coping tactics, such as mindfulness techniques, breathing exercises, and positive imagery. Furthermore, introducing emotional intelligence into music education, such as training students to comprehend and express their feelings through music, can help them improve their emotional comprehension and management abilities.
- Peer support among musicians, particularly in ensemble settings, can greatly minimize performance anxiety. Peer encouragement and empathy foster a sense of belonging while reducing feelings of isolation and rivalry, both of which are frequently linked to performance anxiety. Sharing experiences and coping skills with peers can also assist to normalize performance anxiety and provide practical solutions for managing it. Fostering a collaborative and supportive environment during group performances might enable a focus on community accomplishment rather than individual fear of failure, so minimizing anxiety.
- Music schools and departments can establish comprehensive support programs that include the roles of parents, teachers, and peers in treating performance anxiety. These programs may include lectures for parents on how to support their child’s musical journey, teacher training on incorporating emotional intelligence and anxiety management into their instruction, and peer mentorship or support groups for students. Such programs can establish a comprehensive support system that targets both the development of musical talents (self-efficacy) and the emotional components (emotional intelligence) of performance.

### 4.2 Limitations and prospects for future research

Cross-sectional studies capture data at a certain point in time. Because there is no data on the evolution of variables over time, cross-sectional research cannot show the trajectory of development or the potential impact of individual changes in social support on music performance anxiety across a student’s university experience. To solve this restriction, it is recommended to use a longitudinal study design. Longitudinal studies allow researchers to track the evolution and variations of social support, self-efficacy, and emotionality among university students across time. These investigations also look into the link between these alterations and variations in music performance anxiety.

Self-reported metrics will always be subjective. The tendency for people to portray themselves in a positive light, whether they are conscious of it or not, is known as social desirability bias. This can lead to people overestimating or underestimating psychic health or emotional intelligence. Furthermore, self-reported statistics rely on the individual’s ability to recall and accurately record feelings, behaviors, or experiences. This recall may not be perfect, which could indicate that the information is incorrect. In order to overcome this constraint, it is recommended to add self-reported data using qualitative techniques such as focus groups and in-depth interviews.

## 5 Conclusion

The objective of this study was to examine the influence of social support, self-efficacy, and emotional intelligence on music performance anxiety among undergraduate and postgraduate students in China. By conducting thorough investigation, which involved SEM and multigroup analysis, we discovered several significant insights. First and foremost, receiving social support from parents, teachers, and peers has a substantial impact on the improvement of both self-efficacy and emotional intelligence. These personal abilities are essential for reducing music performance anxiety. This suggests that the presence of a nurturing atmosphere around music students is crucial for their emotional health and ability to succeed.

The findings indicate that these impacts are more prominent among undergraduate students in comparison to postgraduate students. This discrepancy indicates that younger students, who may be in the initial phase of their academic and personal growth, derive greater advantages from external support systems. Undergraduates are very dependent on parental and educational assistance as they manage the difficulties of higher education and the stress of academic excellence.

The take-home message from this study is that it is essential for music students to cultivate strong support networks and enhance personal competences, such as self-efficacy and emotional intelligence, in order to decrease performance anxiety. These individual abilities act as intermediaries in the connection between social support and music performance anxiety, underscoring the significance of a comprehensive strategy to supporting students. It is crucial for educational institutions and families to prioritize offering continuous and significant assistance to students, especially throughout their college years, in order to enhance their emotional well-being and music performance.

## Data Availability

The raw data supporting the conclusions of this article will be made available by the authors, without undue reservation.
