# Supplementary material for: Effects of social support on music performance anxiety among university music students: chain mediation of emotional intelligence and self-efficacy
Source: Front Psychol. 2024 Sep 17;15:1389681. doi: 10.3389/fpsyg.2024.1389681 (PMC11457729; doi:10.3389/fpsyg.2024.1389681)
Supplement: Supplementary file 1 [file Table_1.DOCX]

**Power Analysis**

The significance of sample size determination using power analysis to guarantee the reliability and validity of study findings has been underscored by recent advancements in research methodology (Kline, 2010; Ringle, Sarstedt, Mitchell, & Gudergan, 2020; Uttley, 2019). Power analysis is a statistical method employed to determine the least magnitude of the sample size needed for research, so assuring that it possesses the statistical power to identify any potential effect. This is especially crucial in research where the intricacy of the model, such as those including a substantial number of predictors, can impact the required sample size (Kang, 2021). Power analysis enables researchers to quantify the sample size required to identify statistically significant results by considering variables such as power, effect size, and significance level.

Power is a statistical measure that quantifies the probability of a statistic properly rejecting the null hypothesis when it is actually untrue (Wang & Rhemtulla, 2021). Consequently, power is a measure of the probability of correctly detecting a real effect, and a power level of 80 percent or more is seen sufficient in social scientific research (Cohen, 2013; Uttley, 2019). Attaining this degree of statistical power guarantees that the research is sufficiently strong to identify significant effects and minimizes the likelihood of Type II errors, which involve the erroneous acceptance of a false null hypothesis.

The effect size is a crucial element in power statistical analysis. The effect size hypothesis quantifies the extent to which the independent factors influence the dependent variable (Myors, Murphy, & Wolach, 2010; Sullivan & Feinn, 2012). Gaining a comprehensive understanding of the effect size is crucial in order to accurately estimate the necessary sample size. Typically, bigger effect sizes need smaller sample sizes to attain the needed statistical power, while lower effect sizes require larger samples. In order to get a statistical power of 80 percent or above, it is crucial to assess the anticipated effect size, which can often be approximated by referring to effect sizes documented in other studies on comparable subjects. The general benchmarks for evaluating effect sizes were established by Cohen (2013), who proposed that values of 0.02, 0.15, and 0.35 are indicative of small, medium, and large effects, respectively.

The significance level (α) is a critical parameter in power analysis that quantifies the likelihood of rejecting the null hypothesis when it is indeed true. A significance level of 0.05 (5% equivalent) is generally regarded as the standard threshold in social and behavioral sciences (Hair, Black, Babin, & Anderson, 2010). This level denotes a 5% probability of making a Type I error, which is the erroneous rejection of the null hypothesis. Using the suitable power level, effect size, and significance level, researchers can precisely ascertain the minimal sample size required to guarantee that the results of their study are statistically significant and scientifically relevant. This methodology helps to prevent research with insufficient statistical power that may fail to discover genuine effects or studies with excessive statistical power that needlessly consume more resources than necessary.

Figure 1 shows the power analysis with G*Power.


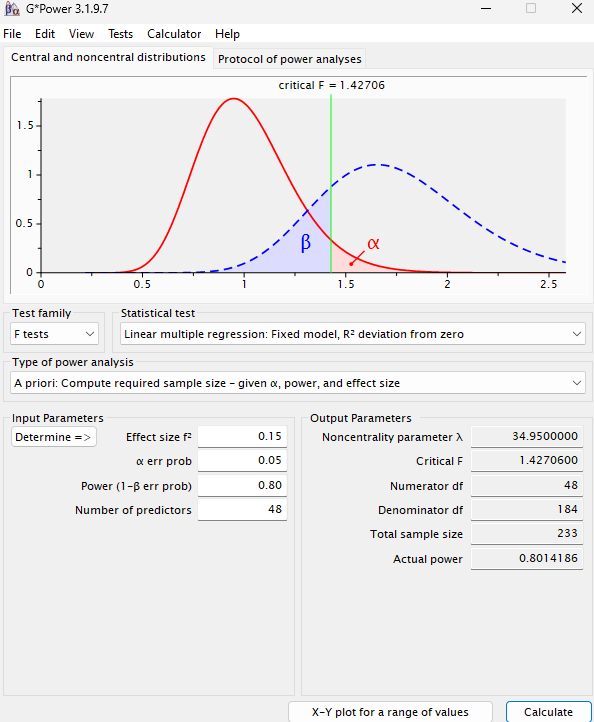


Figure 1. G*power outputs

The outputs indicate that a minimum of 233 samples is required to achieve adequate statistical power for the analysis. However, since the study involves comparing two distinct groups — postgraduate and undergraduate students — the sample size must be doubled to ensure sufficient representation for both groups. Therefore, a total of 466 students (233 from each group) is needed to conduct this study effectively, allowing for meaningful comparisons and valid conclusions across the two educational levels.

References

Cohen, J. (2013). *Statistical power analysis for the behavioral sciences*: routledge.

Hair, J. F., Black, W. C., Babin, B. J., & Anderson, R. E. (2010). Multivariate data analysis: A global perspective. *Pearson: Upper Saddle River, NJ*.

Kang, H. (2021). Sample size determination and power analysis using the G* Power software. *Journal of educational evaluation for health professions, 18*. doi:<https://doi.org/10.3352/jeehp.2021.18.17>

Kline, R. B. (2010). *Principles and practice of structural equation modeling*: Guilford press.

Myors, B., Murphy, K. R., & Wolach, A. (2010). *Statistical power analysis: A simple and general model for traditional and modern hypothesis tests*: Routledge.

Ringle, C. M., Sarstedt, M., Mitchell, R., & Gudergan, S. P. (2020). Partial least squares structural equation modeling in HRM research. *The International Journal of Human Resource Management, 31*(12), 1617-1643. doi:<https://doi.org/10.1080/09585192.2017.1416655>

Sullivan, G. M., & Feinn, R. (2012). Using effect size—or why the P value is not enough. *Journal of graduate medical education, 4*(3), 279-282. doi:<https://doi.org/10.4300/JGME-D-12-00156.1>

Uttley, J. (2019). Power analysis, sample size, and assessment of statistical assumptions—Improving the evidential value of lighting research. *Leukos*. doi:<https://doi.org/10.1080/15502724.2018.1533851>

Wang, Y. A., & Rhemtulla, M. (2021). Power analysis for parameter estimation in structural equation modeling: A discussion and tutorial. *Advances in Methods Practices in Psychological Science, 4*(1), 2515245920918253. doi:<https://doi.org/10.1177/2515245920918253>
